# Fluoride exposure among children with different blood groups at Kolar district, Karnataka, India

**DOI:** 10.6026/973206300200234

**Published:** 2024-03-31

**Authors:** Kurpad Nagaraj Shashidhar, Anantharamaiah Hemalatha, Uppalamethi Munilakshmi, Paramaturi Sharon Rose, Muthyala Meghanath

**Affiliations:** 1 Department of Biochemistry, Sri Devaraj Urs Medical College, Kolar, Karnataka, India

**Keywords:** Fluoride exposure, children, blood groups, Kolar district, Karnataka

## Abstract

Fluoride is present naturally in water at < 1.5 ppm and it is considered to be essential for dental and bone enamel mineralization forming fluorapatite. Chronic exposure (> 2ppm) is considered to be toxic and it leads to fluorosis. Fluoride in minor quantities is excreted through sweat, saliva and feaces. Kidneys are the major route of excretion of fluoride and are thus it is the primary organ to be affected. Therefore, it is of interest to assess urinary fluoride among school children with different blood groups at Kolar district, Karnataka, India. Study was conducted in 155 subjects, aged 16-17 years. Data shows that blood group B has high risk of effecting by fluorosis (mean value of urine fluoride = 1.11). However, subjects with negative blood groups are least affected by fluorosis (mean value of urine fluoride =0.30). However, a larger population study is required for validation.

## Background:

Fluoride is derived from Fluorine, the 13th most abundant element on earth's crust [1]. It is present in majority of foods and beverages including water [2]. Fluoride levels vary among the population [3]. Fluoride accumulation of ground water varies according to the source of water, geological formulation of the area and quantum of rain fall [3]. Fluoride in permissible limits protects against dental caries in children [4]. Excess intake of fluoride results in adverse events and health hazards [5]. Direct resultants of chronic fluoride exposure are Dental Fluorosis, Skeletal Fluorosis and Non-Skeletal fluorosis [6]. Further excessive intake of fluoride indirectly impairs the Cognitive, Renal and other physiological functions [7, 8-9]. Kolar district of Karnataka is a fluoride endemic area [10]. Underground drinking water of this area is rich in Fluoride. Healthy range of fluoride in drinking water is upto 1.5ppm [11]. Studies and population surveys suggest the mean value of fluoride levels of Kolar district is around 3.06ppm, substantiating the adverse events of high fluorosis exposure [12]. Therefore, it is of interest to assess the effect of fluoride exposure among school children with different blood groups in India.

## Materials and Methods:

This is an observational study conducted in a school health education camp at Kolar district. A total of 155 subjects of were enrolled with age group of 16-17 years. Anthropometric measurements, Blood pressure, Urine fluoride and blood group categorization was done to the study subjects. Around 25 ml of urine was collected from each subject. Urine Fluoride was determined using Thermo Scientific Ion selective electrode and Total Ionic Strength Adjustment Buffer (TISAB) II buffer. TISAB was used to provide a constant background ionic strength that de-complexes fluoride ion and adjusts the pH of the sample. Chemicals and reagents used were of analytical grade. Blood Grouping was done through ABO typing. Selected subjects were categorized by gender and blood group. Mean values of anthropometric measurements, Systolic blood pressure SBP, Diastolic blood pressure DBP and urine fluoride were measured and statistically analyzed.

## Results and Discussion:

Kolar district is an endemic area for fluoride with scanty rainfall and has affected at large [12]. Local population including agriculture, Floriculture, Horticulture, Livestock and various other agricultural dependencies are also not spared. Correlation of Urine fluoride with anthropometric measurements including BMI calculation and BP measurements was carried out. Pearson's correlation depicted a notable correlation of urine fluoride with systolic blood pressure (SBP) with p value of -0.007 (Table 1). There is no significance observed among the correlation of urine fluoride and blood groups (Figure 3 - see PDF). Gender based comparison of urine fluoride between males and females with a total number of 155 study subjects were analyzed (Figure 1 - (see PDF) and Figure 2 - (see PDF)). The same was tabulated in Table 2. We could observe a positive correlation with urine fluoride, SBP and DBP. However, the BMI and urine fluoride did not show any statistical significance. Correlation of urine fluoride with blood groups showed predominantly 24 male with B+ blood group and 22 with O+ blood group; females we could observe equal distribution of 14 in both B positive and O positive, respectively (Figure 1 - (see PDF) and Figure 2 - (see PDF)). The other blood groups viz O-, A+, A-, B-, AB+ and AB- were less in number compared to B+ and O+ respectively (Table 4). It has been documented that Genetics play a critical role in determination of blood groups [13, 14]. However the impact of fluoride on alleles needs to be determined, as studies conducted by Marganwar *et al.* observed that high fluoride concentration may disturb the anion channel of erythrocytes membrane, which in turn leads to haemolysis and swelling of cells. This causes toxic effect on RBC's and their metabolomics and Epigenetics [15]. Scattered diagram with comparison of urine fluoride versus blood group as R value of 0.02 indicated a non-significance of fluoride on blood groups. However a large population studies with Epigenetic studies impart the predetermined effects of fluoride on the blood group and RH and also blood homeostasis. We could observe >1ppm in the blood groups B, O, A and AB with highest to lowest of 18, 16, 12 and 2 (Table 3). In a cross-sectional study conducted by Ali Al Ghamdi, concluded that people with Blood group B are at higher risk of developing severe forms of Periodontitis [16]. Our study strengthens his point as we observed more number of subjects having urine fluoride value of >1ppm in blood group B. However, within the blood group RH positive and negative determined 16/16 of O+, 11 of 12 in A+, 17 out of 18 in B+ and 2 on 2 of AB+ indicating the negative blood groups are comparatively protective against fluorosis and its role (Table 3). Earlier studies of Mazumdar *et al.* excluding negative blood groups, concluded with no statistical significance [17]. We included Rh-ve groups and a larger population than those and submitting an output that negative blood groups are better protected against fluorosis. Haematological studies concluded that red blood cell antigens are inherited traits; they are usually not altered throughout the life of an individual. There have been occasional case reports of ABO blood group antigen change in malignant conditions [18].

Awareness programs on fluorosis and its impact prevents the adverse events of highly exposed population [19]. Despite use of RO filters, the filtered out effluent of the water with a high fluoride content discharged onto the ground gets recharged and further concentrates the fluoride in soil leading to further high fluoride of soil and grass [20]. Consumption of the same by poultry and livestock with a resultant consumption of poultry products and milk by humans leads to indirect worsening of the fluorosis in highly fluoridated areas [21, 22]. Studies conducted by Helen G Jarvis and his team suggests providing safe and clean piped water, we too recommend the Government agencies and statutory bodies to take appropriate measures to reduce the salinity of water and soil [23]. Safe disposal of filtered out fluoridated water is to be considered as a Biohazard. We also recommend that RO filters also filters out in addition to fluoride various other essential minerals which are required for enzyme activities and metabolomics, leading to a direct or indirect effect on the Epigenetics and metabolism of fluoride [24, 25].

## Conclusion:

Data shows that blood group B has high risk of effecting by fluorosis. However, subjects with negative blood groups are least affected by fluorosis in this sample size.

## Limitations of our study:

This study is conducted in a fluoride endemic area with a small sample size so to know the impact in correlation with urine fluoride among children with different blood groups. The same is required with a large sample size among different age groups along with serum fluoride.

## Figures and Tables

**Table 1:** Correlation of Urine Fluoride (UF) with Anthropometric measurements and Physiological variables

	**R value**	**Age**	**Gender**	**Blood Group**	**BMI (kg/m^2^)**	**SBP (mm/hg)**	**DBP (mm/hg)**
UF		0.075	-0.056	0.043	-0.047	-0.007*	0.031
	P value	0.351	0.486	0.653	0.573	0.935	0.719
SBP: Systolic blood pressure; DBP: Diastolic blood pressure;
*p-value ≤ 0.05 is considered as statistically significant.

**Table 2:** Gender based comparison of Urine fluoride: (Male: 91; Female: 64)

**Variables**	**Groups**	**Mean ± SD**	**Std. Error Mean**	**P Value**
Age (in years)	Male	16.6 ±0.52	0.05	0.02*
	Female	16.70 ± 0.46	0.05	
Blood	Male	3.52 ± 2.03	0.23	0.24
Group	Female	3.05 ± 1.92	0.32	
BMI	Male	21.02 ± 4.69	0.49	0.81
(kg/m^2^)	Female	21.20 ± 4.31	0.56	
SBP	Male	113.45 ± 13.39	1.46	0
(mm/hg)	Female	111.37±11.85	1.57	
DBP	Male	68.85 ± 10.44	1.13	0.02*
(mm/hg)	Female	72.61 ± 9.36	1.24	
Urine Fluoride	Male	1.14 ± 0.41	0.004	0.48
(ppm)	Female	1.09 ± 0.43	0.05	

**Table 3:** Study subjects (155) were categorized into two groups based on Urine Fluoride value (<1ppm and >1ppm). With this categorization, UF values are correlated with BMI, SBP, DBP and blood groups to find out the p value

**UF**	**O**	**O+ve**	**O-ve**	**A**	**A+ve**	**A-ve**	**B**	**B+ve**	**B-ve**	**AB**	**AB+ve**	**AB-ve**
<1 ppm N = 65	23	20	3	12	12	0	23	21	2	5	3	2
>1 ppm N = 90	16	16	0	12	11	1	18	17	1	2	2	0

**Table 4:** Correlation of Urine fluoride with Blood groups

**Chi-square value**	**BMI**	**SBP**	**DBP**	**O+ve**	**A+ve**	**B+ve**	**O (+ & -)**	**A (+ & -)**	**B (+ & -)**	**AB (+ & -)**
	6.95	0.125	0.35	2.19	0.85	2.19	3.65	0.55	2.62	2.37
*p-value*	0.00836*	0.723	0.54	0.13	0.35	0.13	0.056*	0.45	0.1	0.123
We have considered the significance with p-value ≤ 0.05,
significance of Urine-fluoride is observed only with BMI and blood group O.
